# Spontaneous intracranial hypotension in a patient without corpus callosum: A case report

**DOI:** 10.1097/MD.0000000000039090

**Published:** 2024-07-26

**Authors:** Yang Zhou, Chenglin Tong

**Affiliations:** a Department of Neurology, Shaoxing Second Hospital, Shaoxing, Zhejiang, China; b Department of Emergency Internal Medicine, Shaoxing Second Hospital, Shaoxing, Zhejiang, China.

**Keywords:** case reports, corpus callosum agenesis, headache, spontaneous intracranial hypotension

## Abstract

**Rationale:**

Spontaneous intracranial hypotension (SIH) is a well-established condition typically presenting with disabling orthostatic headache. Corpus callosum agenesis (CCA) is one of the most common human brain malformations with a wide spectrum of associated malformations, chromosomal abnormalities, and clinical syndromes.

**Patient concerns:**

A 53-year-old woman presented with recurrent orthostatic headache for about 1 month. The head computed tomography examination of the patient showed CCA and the initial pressure of subsequent lumbar puncture was only 5 centimeters cerebrospinal fluid. Magnetic resonance imaging examination confirmed CCA with gray matter heterotopia.

**Diagnosis:**

The final diagnose was SIH related headache with CCA.

**Intervention:**

The patient’s symptom improved after oral hydration, intravenous fluids, and bed rest.

**Outcome:**

Favorable outcome was observed.

**Lessons:**

Although this co-occurrence of SIH and CCA is probably coincidental, this finding suggests that the developmental malformations of the brain may lead to structural changes in brain tissue or disturbances in cerebrospinal fluid production and reflux, resulting in pathological changes over time.

## 1. Introduction

Though once considered a rare disease, a considerable rise in interest in spontaneous intracranial hypotension (SIH) is seen in recent times, with many additional cases having been published. The typical presentation of SIH is intracranial hypotension related orthostatic headache. Corpus callosum agenesis (CCA) forms a failure to develop the large bundle of fibers that connect the cerebral hemispheres and a disruption in interhemispheric connections. CCA induces a range of abnormalities in brain structure and function, mostly psychiatric disorders, cognitive decline, and epilepsy, some rare cases are also present such as bilateral degenerative changes and alien limb syndrome in Parkinson disease patients,^[1,2]^ CCA together with congenital lymphedema and other hereditary disorders.^[3,4]^

In this article, we present a patient diagnosed with SIH in whom the images revealed CCA. Both SIH and CCA affect the brain and may cause neurological symptoms, but there is no known causal relationship between the 2 conditions. Whether the onset of intracranial hypotension is related to the absence of the corpus callosum or is independent, needs to be further studied.

## 2. Case report

**Figure 1:**
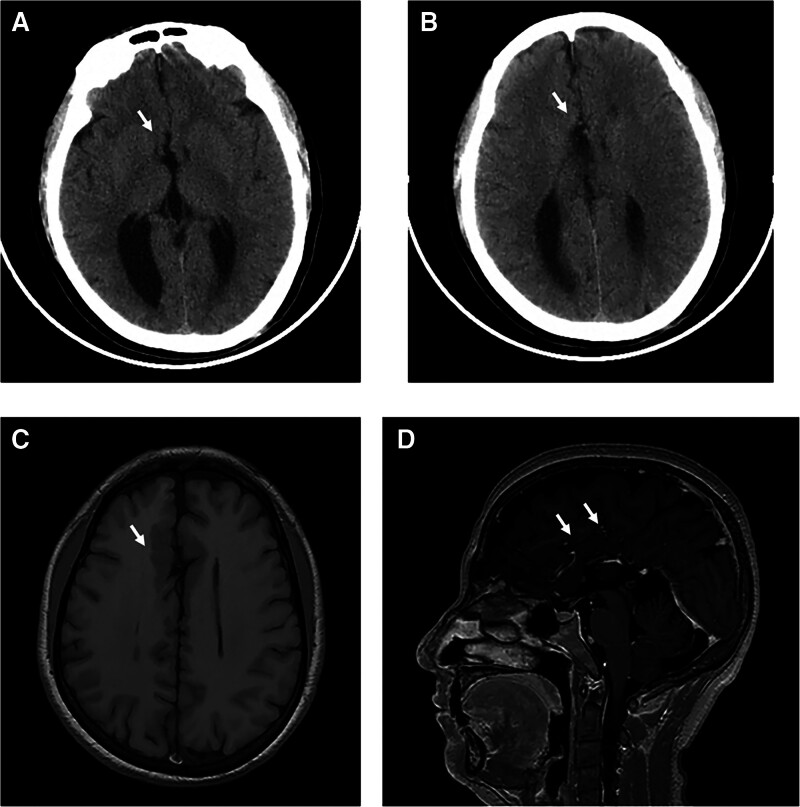
. Corpus callosum agenesis. (A, B) Axial noncontrast CT showed corpus callosum agenesis (white arrowheads). Axial T1-weighted image without contrast (C) and sagittal contrast-enhanced T1-weighted image (D) confirmed the agenesis of corpus callosum with gray matter heterotopia (white arrowheads). CT = computed tomography.

**Figure 2:**
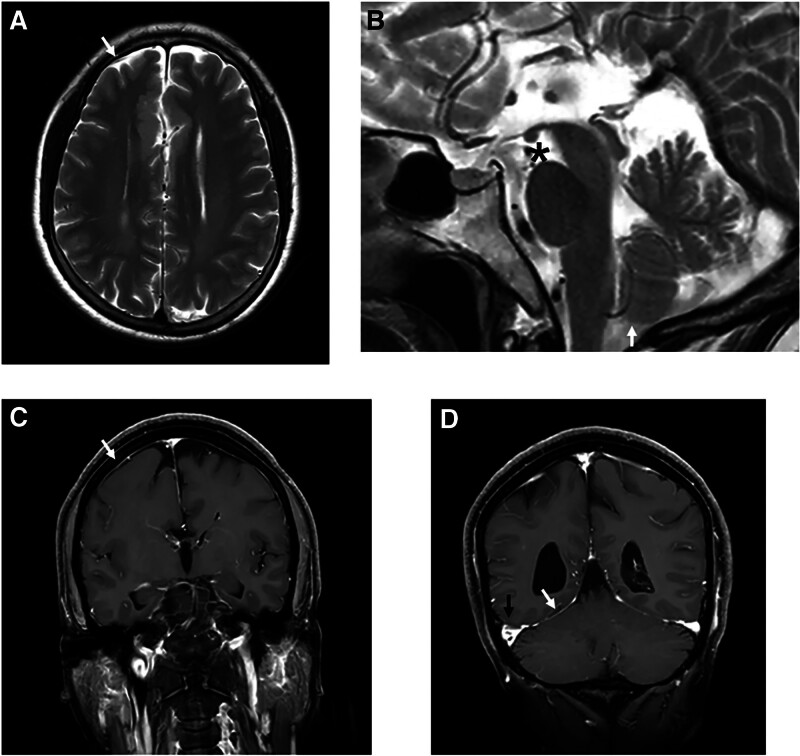
. Imaging findings of spontaneous intracranial hypotension. (A) Axial noncontrast T2-weighted image with subdural effusion (white arrowhead). (B) Sagittal noncontrast T2-weighted image with decreased mamillopontine distance (asterisk: 5.43 mm, pathologic ≤ 6.5 mm) and low-lying cerebellar tonsils (white arrowhead). (C, D) Coronal contrast-enhanced T1-weighted image with slightly dural enhancement (white arrowheads) and venous engorgement (black arrowhead).

**Figure 3:**
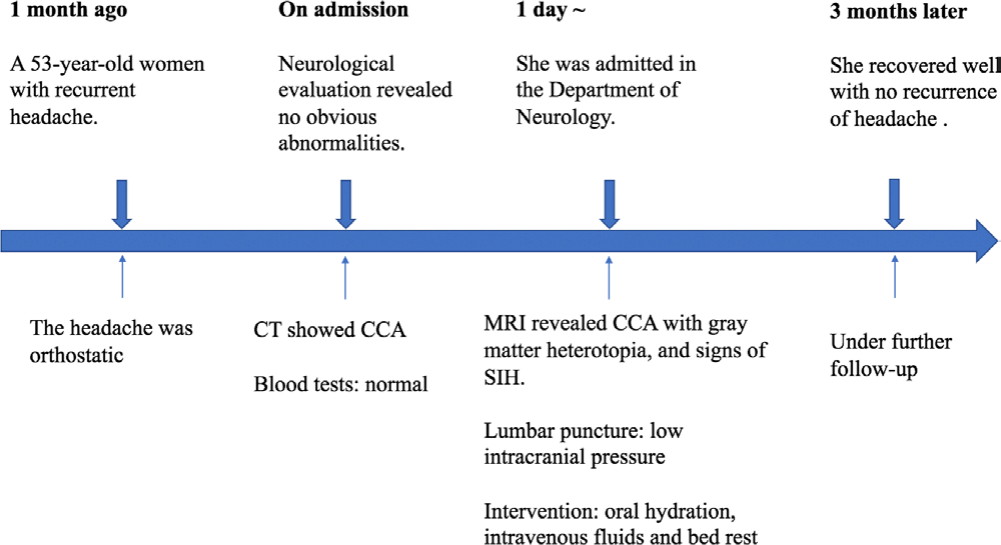
. Timeline of the case.

A 53-year-old woman presented to our hospital with a complaint of recurrent headache for about 1 month. She was unable to recall a history of similar headaches, and had no family history of underlying medical conditions, developmental delays, or genetic disorders. The headache was orthostatic, localized in the posterior occipital region, and occurred and worsened after assuming an upright position, but improved after lying down. She was conscious, well-oriented, and had normal pulse with regular rhythm and normal blood pressure. Neurological evaluation revealed no obvious abnormalities. No significant abnormalities were found in the complete blood count, glucose tolerance test, lipid profile, liver, kidney, and thyroid function tests, coagulation screen, and venereal disease tests. The head computed tomography (CT) examination of the patient on admission showed CCA (Fig. 1A and B). Subsequent lumbar puncture in supine position was perfected, and the initial pressure was only 5 centimeters cerebrospinal fluid (CSF). Nucleated cells, erythrocytes, glucose, protein, gram stain, and cultures of her CSF were all normal. The next day’s magnetic resonance imaging (MRI) examination confirmed CCA with gray matter heterotopia (Fig. 1C and D). Coronal T2-weighted MRI showed a small amount of convex subdural effusion, sagittal T2-weighted MRI revealed decreased mamillopontine distance and low-lying cerebellar tonsils, enhanced T1-weighted MRI showed dural enhancement and venous distension (Fig. 2), consistent with MRI signs of SIH.^[5]^ However, the patient’s symptom improved after oral hydration, intravenous fluids and bed rest. The patient refused invasive tests such as myelography to look for the cause of low intracranial pressure or CSF leak. Three months after discharge, she complained of no recurrence of headache and was still under further follow-up. Timeline of this patient was shown in Figure 3.

## 3. Discussion

CCA is a congenital condition in which the corpus callosum, the thick bundle of nerve fibers connecting the 2 cerebral hemispheres, fails to develop fully or is entirely absent. This can happen during fetal development and is often detected early in life or during brain imaging for unrelated issues.^[6]^ The corpus callosum plays a crucial role in facilitating communication between the left and right hemispheres of the brain. CCA can lead to various neurological and cognitive symptoms, which can vary depending on the extent of the agenesis and any associated brain abnormalities. It is associated with a large variability in psychological, developmental, cognitive and behavioral symptomatology,^[7]^ the patient has no psychiatric disorders, seizure, or cognitive decline, but has been confirmed to have intracranial hypotension headache. SIH is a condition characterized by low CSF pressure within the skull, resulting in various symptoms.^[8]^ The most common identifiable cause of SIH is the spontaneous leakage of CSF from the dural sac, which surrounds the spinal cord and brain.^[9]^ The diagnosis of SIH is based on clinical symptoms, brain imaging and sometimes additional tests to confirm CSF leakage, like radioisotope cisternography.^[10,11]^ Treatment of SIH usually involves conservative measures such as bed rest, hydration, and pain relief. In some cases, an epidural blood patch may be performed to seal the CSF leak and restore normal pressure.^[12]^ The mechanism underlying headache is presumed to the sagging of cerebral structures and traction or distortion of pain-sensitive nerves ending in the cranial dura and its vasculature. In this case, the sagging and downward displacement of the brain tissue might pull the meningeal pain fibers, compress nearby veins such as the Galen and the straight sinus, lead to the venous blood flow stasis and engorgement, causing headache and these corresponding imaging findings. However, the patient was discharged after infusion treatment and no further examination was conducted to investigate the cause of SIH. According to Monro–Kellie doctrine, in order to keep the intracranial pressure constant, the volume loss of brain tissue is compensated by an increase of the volume of the other compartments, however, this compensatory capacity is limited with aging,^[13]^ this may explain why the patient recently developed the headache. CCA has not been reported in cases with SIH. Although both SIH and CCA can affect the brain and may lead to neurological symptoms, there is no known causal relationship between these 2 conditions. Whether the attack of intracranial hypotension is related to the absence of corpus callosum in the patient, or it is independent. Based on the prevalence of SIH and CCA, and the possible causes of both entities, the concurrence of these 2 diseases in this patient was probably coincidental. The pathophysiology of SIH and the related headache in the patient without corpus callosum remains presumptive and further studies are needed.

## 4. Conclusion

The presented case shows a patient without corpus callosum diagnosed with SIH related headache, although the occurrence of these 2 may be accidental, this finding suggests that CCA might lead to the developmental sinking of brain tissue or abnormal production and reflux of CSF, theoretically this correlation is based on temporal coincidence and the importance from structural development to functional abnormality should be underlined.

## Author contributions

**Conceptualization:** Yang Zhou.

**Investigation:** Yang Zhou.

**Writing – original draft:** Yang Zhou.

**Writing – review & editing:** Yang Zhou, Chenglin Tong.

**Data curation:** Chenglin Tong.

**Supervision:** Chenglin Tong.

**Validation:** Chenglin Tong.
